# Biomineralization Guided by Paper Templates

**DOI:** 10.1038/srep27693

**Published:** 2016-06-09

**Authors:** Gulden Camci-Unal, Anna Laromaine, Estrella Hong, Ratmir Derda, George M. Whitesides

**Affiliations:** 1 Department of Chemistry and Chemical Biology, Harvard University, 12 Oxford Street, Cambridge, MA 02138, USA; 2 Institut de Ciència de Materials de Barcelona, ICMAB-CSIC, Campus UAB, Bellaterra, Catalunya, E-08193 Spain; 3 Department of Chemistry, University of Alberta, Edmonton, Alberta, T6G 2G2, Canada; 4 Wyss Institute for Biologically Inspired Engineering, Harvard University, 60 Oxford Street, Cambridge, MA 02138, USA

## Abstract

This work demonstrates the fabrication of partially mineralized scaffolds fabricated in 3D shapes using paper by folding, and by supporting deposition of calcium phosphate by osteoblasts cultured in these scaffolds. This process generates centimeter-scale free-standing structures composed of paper supporting regions of calcium phosphate deposited by osteoblasts. This work is the first demonstration that paper can be used as a scaffold to induce template-guided mineralization by osteoblasts. Because paper has a porous structure, it allows transport of O_2_ and nutrients across its entire thickness. Paper supports a uniform distribution of cells upon seeding in hydrogel matrices, and allows growth, remodelling, and proliferation of cells. Scaffolds made of paper make it possible to construct 3D tissue models easily by tuning material properties such as thickness, porosity, and density of chemical functional groups. Paper offers a new approach to study mechanisms of biomineralization, and perhaps ultimately new techniques to guide or accelerate the repair of bone.

Bone serves multiple functions in vertebrates: it provides a mechanical structure, anchors muscles, stores minerals, and provides a compartment in which red and white blood cells form^1^. Disease, degeneration, aging, or trauma cause loss or damage to bone in the body^2^,^3^. While there have been clear improvements in generation of grafts for bone, it remains difficult to fabricate porous and biocompatible scaffolds in sizes (cm-scale) relevant to clinical practice^4^. Scaffolds for bone have been fabricated using different methods, including 3D printing, electrospinning, gas foaming, injection molding, and salt leaching^5^. These methods require special equipment, extensive optimization procedures, and trained personnel. Inexpensive, easy-to-use, and widely accessible materials capable of templating mineralization would, therefore, be useful for developing new scaffolds for bone. When combined with sources of cells that are compatible with a host, these scaffolds have the potential (in the future) to contribute to the healing of bone.

Currently available synthetic scaffolds are not biocompatible without the incorporation of biological cues (e.g., functional groups that interact with cells, growth factors, small molecules, and the presence of minerals)^5^. Another major problem with existing bone scaffolds is the inability to promote vascularization within the scaffold^4^. To increase the biological functionality and vascularization potential of the scaffolds, signaling molecules such as bone morphogenetic protein (BMP-2), vascular endothelial growth factor (VEGF), and fibroblast growth factor (FGF-2) have been incorporated into scaffolds^6^,^7^,^8^. Scaffolds that contained inorganic minerals were also used to induce mineralization^9^. Another important limitation with current scaffolds is the absence of cellular components. Because non-cellular scaffolds do not easily integrate with the tissue when implanted in the body^10^, strategies for cellularization have therefore been developed to improve the osteointegration of synthetic grafts of bone^5^. A summary for the major drawbacks of currently available scaffolds is given in Tables S1 and S2. Paper is an alternative material to address the major limitations of conventional scaffolds (e.g. commercial unavailability, high-cost, low porosity, inflexibility/rigidity, biocompatibility, untunability, and requirement of sophisticated fabrication procedures).

Cellulose, a polysaccharide with natural origin, has been investigated for biomedical applications^11^,^12^,^13^. Cellulose is a biocompatible matter and does not induce inflammation^14^. The material of choice to fabricate scaffolds in this work was paper, a substrate made of cellulose^15^. Although it is a suitable biomaterial for tissue engineering, cellulose has to be processed in order to fabricate scaffolds, which might require long and sequential procedures or optimization of protocols and labor. Compared to cellulose and other biomaterials that are derived from plant materials, paper is a readily and commercially available low-cost material. Whatman paper is pure cellulose fiber, and does not include lignin, binder, brightener, or sizing agents^16^,^17^.

Although they have been explored for other aspects of cell biology^18^,^19^,^20^,^21^,^22^, the simple and flexible features of paper-based materials have not been used to guide deposition of hydroxyapatite by osteoblasts. We previously reported a technique for culturing cells in gel-impregnated paper scaffolds^18^. We refer to this approach as cells-in-gels-in-paper (CiGiP). It provides an experimentally simple method to generate 3D tissues. The CiGiP approach forms multi-layered 3D models of tissues by stacking layers containing cells in the hydrogel slabs supported mechanically on paper. We used CiGiP for studying the migration of mammalian cells^21^, developing invasion assays^19^,^20^, generating parallel arrays of 3D cell cultures, high-throughput testing of drugs for cancer cells^22^, and controlling diffusion of oxygen and nutrients to cells that were cultured in multi-layered stacks^18^. In addition to cancer cells, we also cultured cardiac myocytes, fibroblasts, and primary lung tumor cells in paper^21^,^23^. The cell cultures in paper allowed high cell viability and metabolic activity, as well as biocompatibility upon subcutaneous implantation in mice^18^. The scaffolds made of paper combine the simplicity of a biocompatible material with the ability to form free-standing structures.

We used paper as a scaffold in this work for five reasons: paper is i) readily and universally available; ii) inexpensive, and easily fabricated into 3D shapes; iii) compatible with expansion and growth of osteoblasts (and other cells); iv) easily sterilized; and v) available in a wide variety of porosities, thicknesses, and void volumes. Other paper-like matrices (electrospun fibers) have also been used for generation of scaffolds for bone^24^; however, because of the requirement for an extensive series of optimization steps to obtain the right parameters (molecular weight of the polymer, conductivity, dielectric constant, viscosity, surface tension, temperature, flow rate of the solution, voltage, distance between collector and needle tip) for production of fibers, these materials cannot be readily fabricated.

The paper scaffolds might also demonstrate some disadvantages. Imaging paper by fluorescent microscopy might be difficult for thick paper (e.g. thickness greater than 200 μm) due to light scattering. The mechanical properties of wet paper might demonstrate limitations in impact resistance, toughness, and strength. Paper is not degraded by the enzymes in the body, therefore, it is not a biodegradable material.

## Results and Discussion

### Design and Fabrication of the Paper-Based Platform

There are a wide variety of papers available with structural differences. Whatman filter paper (grade 114) is a commercially available paper that has 190 μm thickness and 25 μm average pore size. We chose to use Whatman 114 filter paper due to its small thickness (no mass transport limitations to diffusion of glucose, oxygen and other nutrients to cells) and sufficiently large pore size to allow for movement of cells through the 3D matrix of fibers. In addition, Whatman 114 is a low-cost and commercially accessible material.

**Figure 1:**
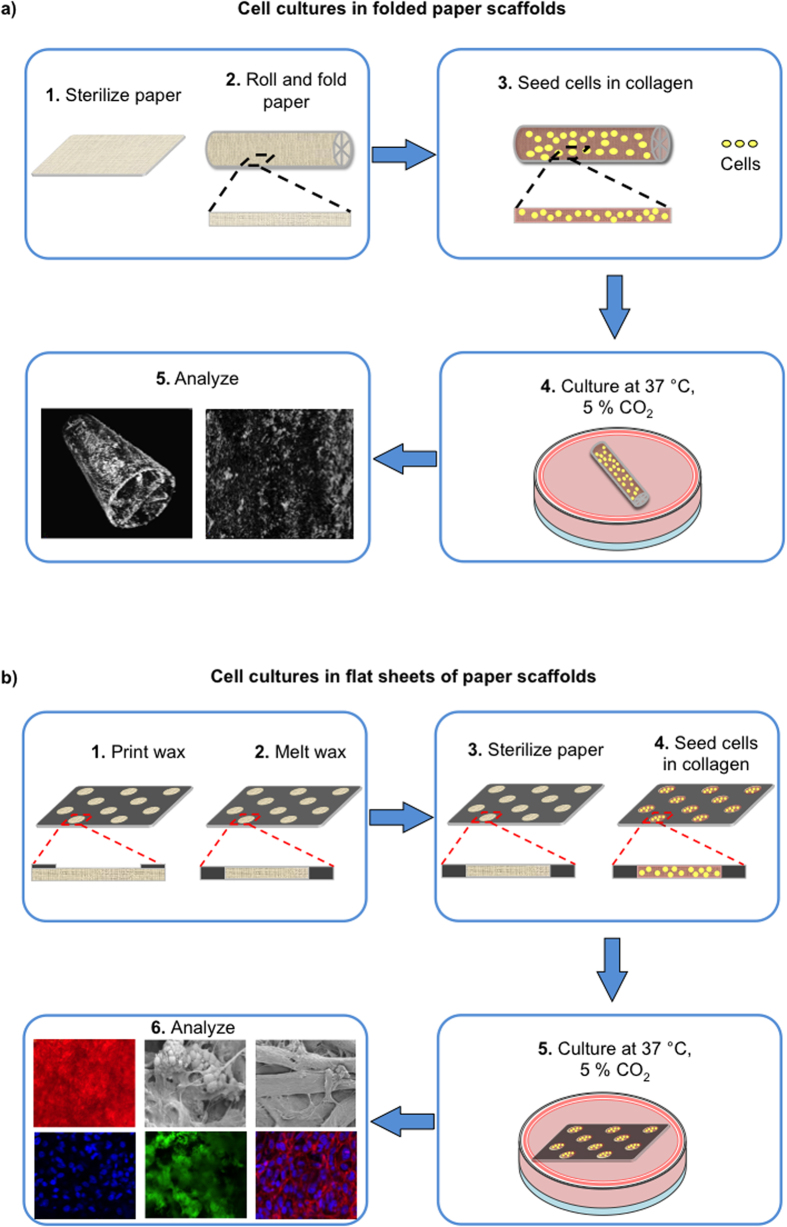
Schematic for the growth of cells on paper scaffolds. We sterilized the paper scaffolds and seeded them with osteoblasts in a collagen matrix. Cells were maintained in position within the thin paper slab upon solidification of collagen. We cultured the cell-encapsulated paper constructs for different periods of time (0, 3, 7, 14, 21 days) at 37 °C with a supplementation of 5% CO_2_ in a tissue incubator. We then analyzed the samples for mineralization. (**a**) Cell cultures in the folded paper scaffolds. We carried out micro-CT analysis to demonstrate the deposition of minerals in the origami-inspired paper constructs. (**b**) Cell cultures in the flat sheets of paper scaffolds. We performed staining for alizarin red, phalloidin/DAPI, hydroxyapatite, and osteocalcin, and also high-resolution imaging (SEM) for the deposition of minerals in the paper scaffolds.

Figure 1 describes the culture of cells on scaffolds fabricated from paper. We used 3D, folded, paper scaffolds as supports for the culture of osteoblasts, and monitored deposition of hydroxyapatite by micro-CT. We used unfolded paper structures, because they were easier to handle than folded structures, and required fewer cells to carry out staining and microscopy analyses. We used collagen type I as the gel matrix as it is an important regulator of bone mineralization and supports the mineralization process^25^.

In the paper scaffolds, cells are included in a collagen matrix. We have not defined whether the cells directly contact the paper or are simply physically included in the gel. The pore size of the paper should be sufficiently large that the cells can migrate in 3D. In this work, we used Whatman filter paper grade 114, which has an average pore size of 25 μm. The size of the osteoblast cells is 10–15 μm, and this pore size should therefore allow for migration of cells. When selecting the type of paper, sufficiently large pore size for infiltration of cells is an important parameter.

The use of paper offers three advantages for 3D culture of osteoblasts. i) It comprises interwoven fibers that resemble, at least morphologically, the fibrillar networks of collagen in bone^26^. ii) Paper has voids between fibers (The filter paper we used has a 25% void volume). This high void volume, the thickness of the paper sheets (190 μm), and the high diffusion constant of low molecular-weight nutrients in collagen gel, are all factors that combine to provide structure that offers the little hindrance to diffusion of nutrients, O_2_, and wastes to/from the cells. The rate of mass transport of oxygen to osteoblasts has been suggested as an important factor determining the rate of mineralization^27^.

### 3D Folded Paper Scaffolds

**Figure 2:**
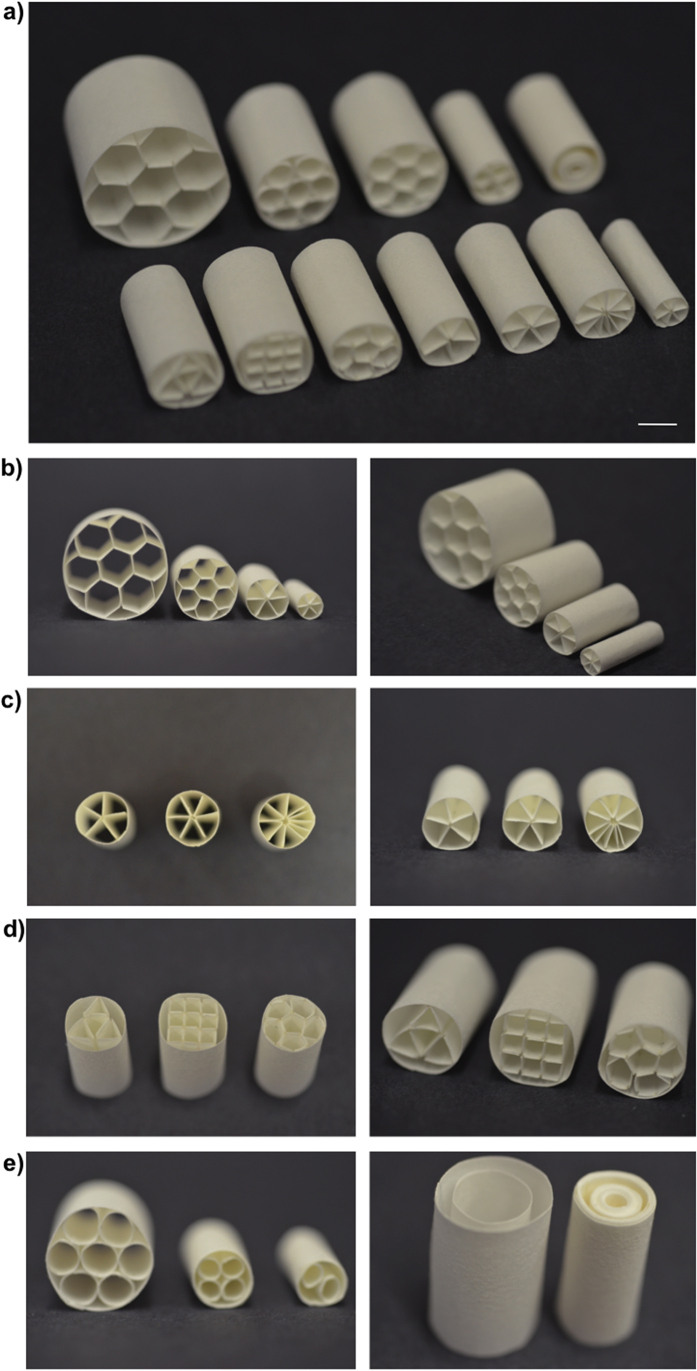
Photographic illustration of the origami-inspired paper scaffolds. (**a**) Paper can easily be cut, creased, and folded to form 3D free-standing constructs. (**b**) The paper scaffolds were fabricated to demonstrate different shape, size, and configuration. (**c**) The paper scaffolds can be shaped into 3D structures including gradient patterns. (**d**) We generated a range of different shapes (e.g., triangle, square, pentagon) in the paper scaffolds. (**e**) We also constructed structures using circular features. Scale bar represents 1 cm.

Paper is a flexible material that can be easily cut, folded, and manipulated to fabricate 3D constructs in a variety of shapes and sizes. We have demonstrated as a proof-of-principle that we can fabricate scaffolds in different shapes (Fig. 2a–e) and we used these shapes primarily to demonstrate possibilities and capabilities. We focused on free-standing structures having cm-scale features (Fig. 2a,b). We generated a number of different types of shapes, including those with circular, triangular, rectangular, pentagonal, hexagonal, and circular features (Fig. 2c–e). The ability to shape paper easily into different structures makes it possible to generate specific architectures, and to fabricate individualized scaffolds that can perhaps, in the future, be designed for patient-specific defects.

### Micro-CT Analysis

**Figure 3:**
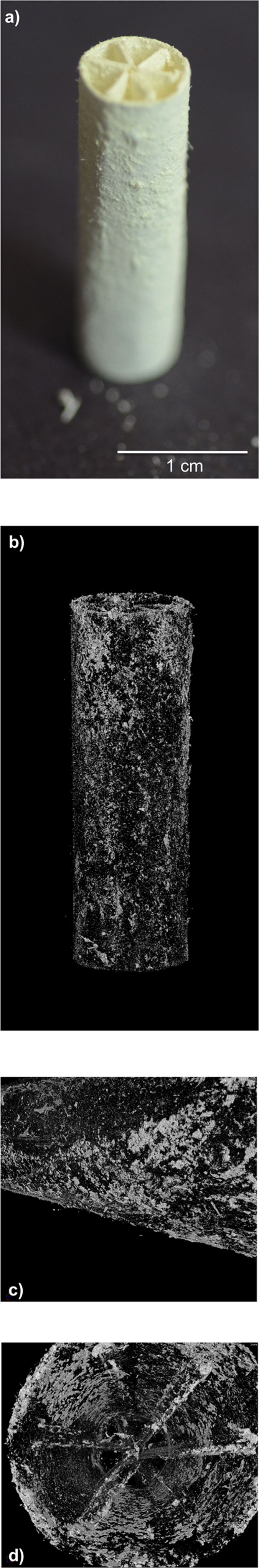
Biomineralized origami-inspired paper scaffold. (**a**) The osteoblasts were seeded in the paper scaffold, cultured for 21 days, and imaged by a single-lens reflex (SLR) camera (Nikon D5100). The construct was mineralized to an extent that was visible to the naked eye. (**b**–**d**) We obtained the micro-computed tomography (micro-CT) X-Ray scans to illustrate the mineralized areas in the paper constructs. The bright white-colored regions in the micro-CT images demonstrate the mineralized areas in the paper scaffolds.

Micro-CT identified the hydroxyapatite phase in the mineralized origami-inspired samples. Micro-CT provides a 3D image of the sample, and is routinely used to map the deposition of hydroxyapatite^28^. After the growth and culture of the osteoblasts for three weeks, we fixed, dried, and photographed the mineralized paper (Fig. 3). The mineralization of the construct was visible to the naked eye (as imaged by a single-lens reflex Nikon D5100 camera). Micro-CT scans then established the distribution of mineralized regions in the paper. The micro-CT images suggest patchy but relatively uniform mineralization (on a scale of 1 cm) (Fig. 3). Approximately 20% volume of the scaffold was mineralized. Figure S1 provides micro-CT images for different geometric configurations (Supplementary Information). These results on biomineralization are promising considering the difficulties associated with mineralization of large (cm-scale) constructs. Partially mineralized scaffolds that include biologically active cells might provide more biocompatible constructs than unmineralized or non-cellular scaffolds^5^. This paper-based system offers a new way to study mineralization, and might lead to new strategies to repair mineralized tissues.

### Deposition of Minerals

**Figure 4:**
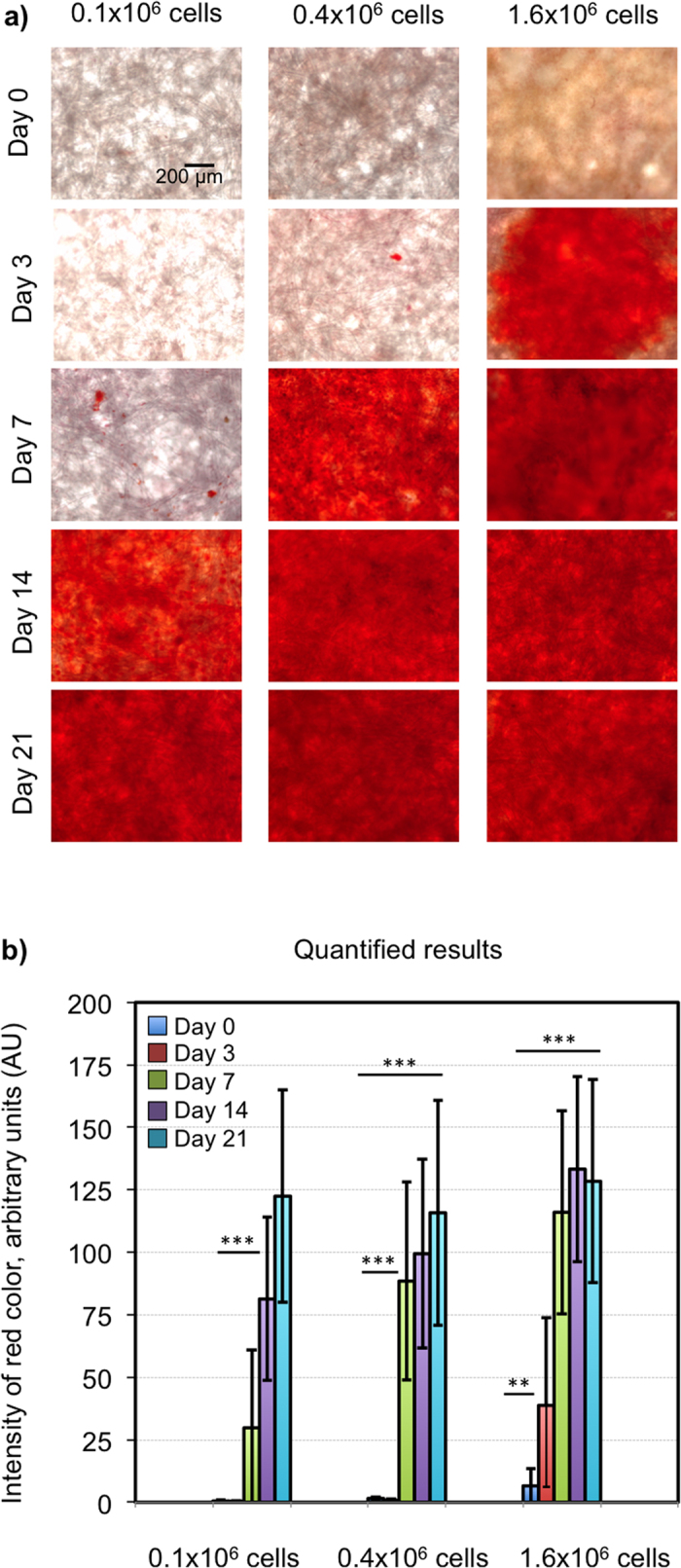
Deposition of bone mineral (calcium phosphate) by osteoblasts was demonstrated by alizarin red staining. Red color is an indication of the reaction between calcium ions and alizarin red dye. (**a**) We seeded the paper at densities of 0.1 × 10^6^, 0.4 × 10^6^, and 1.6 × 10^6^ cells per sample. There was an increase in mineralization as a function of time over 21 days of culture period. The scale bar represents 200 μm. (**b**) We quantified the intensity of red color in the images using the NIH ImageJ software. We analyzed the data using the statistical software GraphPad Prism (Version 4.02, La Jolla, CA). We determined the statistical differences between different conditions using one-way ANOVA analyses (n = 30, Error bars:  ±  SD). We considered *p*-values < 0.05 as statistically significant (**p < 0.01, ***p < 0.001).

We seeded the paper at densities of 0.1 × 10^6^, 0.4 × 10^6^, and 1.6 × 10^6^ cells per sample, and assessed the deposition of the bone mineral (calcium phosphate) microscopically by staining the samples with alizarin red (Fig. 4a); a red color in the images indicates the presence of the complex formed by calcium ions and the alizarin red. Alizarin Red has been a widely used indicator to demonstrate mineralization by cells^29^. NIH ImageJ Software quantified the intensity of red color (Fig. 4b).

When the initial seeding density was low (0.1 × 10^6^ cells/sample), detectable mineralization did not occur until day 14. The samples with moderate cell density (0.4 × 10^6^ cells/sample) began mineralizing at day 7, whereas the samples with high cell density (1.6 × 10^6^ cells/sample) started mineralizing at day 3. Mineralization began after proliferation of osteoblasts and lay-down of extracellular matrix (primarily collagen)^30^. Previous reports suggested that mineralization accelerated as proliferation of cells slowed at higher cell densities^31^.

### Proliferation of Cells

We determined the proliferative activity of the cells on days 0, 3, 7, 14, and 21 (initial cell density: 1.6 × 10^6^ cells/sample) (Figure S2). We stained the nuclei of the cells with DAPI (blue) and imaged them by confocal microscopy. The quantified results of fluorescent staining indicated that proliferation increased until day 3 and then slowed (Figure S2). Because the cells grow to confluence first, and then differentiate^32^,^33^,^34^, a decrease in proliferation is expected to correlate with the beginning of mineralization. The actively proliferating osteoblasts deposit ECM (primarily collagen), which is then progressively mineralized. The correlation between proliferation and deposition of minerals has been described in the literature^30^.

### Hydroxyapatite Content

**Figure 5:**
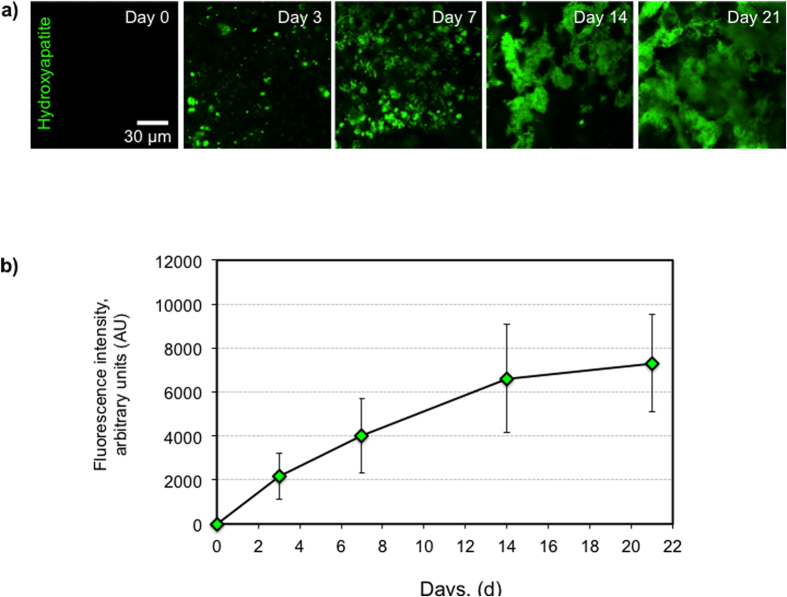
Hydroxyapatite content of the samples. The amount of hydroxyapatite was determined using an OsteoImage assay (Lonza, Walkersville, MD). The fluorescent staining reagent (green) binds to the hydroxyapatite portion of the mineralized matrix, and the fluorescence is measured at 495/519 nm (Ex/Em). The initial cell density was 1.6 × 10^6^ cells/sample. (**a**) The images exhibited green fluorescence proportional to the amount of hydroxyapatite in the samples. (**b**) A progressive increase in the deposition of hydroxyapatite occurred in the paper scaffolds over time (n = 30). The scale bar represents 30 μm.

We assessed the relative amount of hydroxyapatite (calcium phosphate) in the paper-supported cellular composites with a commercial mineralization kit (OsteoImage, Lonza) by measuring the fluorescence at excitation/emission wavelengths of 495/519 nm. This assay, as described by the manufacturer, uses a fluorescent staining reagent that binds specifically to the hydroxyapatite portion of the biomineralized structures^35^. The intensity of the green fluorescence is proportional to the amount of hydroxyapatite in the sample. The initial cell seeding density was 1.6 × 10^6^ cells/sample on the paper scaffolds. There was no signal for mineralization at the beginning of the culture period (on day 0) as expected. Figure 5 exhibits a progressive increase in the amount of hydroxyapatite in the paper scaffolds as a function of time.

### Expression of Bone-Specific Marker by the Cells

We evaluated the expression of a bone-specific marker, osteocalcin—a structural matrix protein, (red) by immunocytochemistry. The initial concentration of cells was 1.6 × 10^6^ cells/sample; the Supplementary Information shows fluorescent images obtained by confocal microscopy (Figure S3). The cells were counter-stained with DAPI (blue) to visualize their nuclei. The expression of the bone-specific protein increased until day 14 during mineralization, and decreased afterwards. We attribute this result to increasing mineralization, and consequent transformation of osteoblasts to osteocytes after day 14. It was previously reported that the decrease in expression of osteolcalcin after mineralization is due to the entrapment of the cells inside the mineralized matrix^36^,^37^. Our results are consistent with this suggestion.

### High-resolution Imaging for Deposition of Minerals

**Figure 6:**
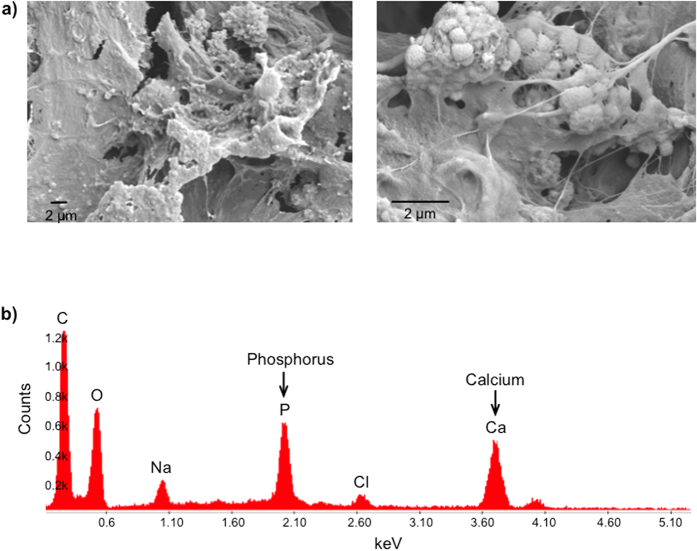
High-resolution imaging for the minerals that were deposited on paper. Scanning electron microscopy (SEM) and energy dispersive X-ray spectroscopy (EDAX) illustrated the presence of calcium and phosphate in the mineralized paper scaffolds.(**a**) The SEM micrographs at different magnifications indicated that mineralized structures formed on the paper constructs after 21 days in culture. The initial seeding density of the cells was 1.6 × 10^6^ cells/sample. (**b**) EDAX analysis confirmed the elemental presence of phosphorus and calcium on the mineralized paper. We determined a Ca:P ratio of 1.5 ± 0.1 from the results of EDAX analysis (n = 7).

We used SEM to image the aggregates of hydroxyapatite and cells on paper. After culturing for 21 days, we fixed, dried, and sputter-coated the samples with palladium/platinum (80:20) prior to imaging at 10 kV (Fig. 6a). We interpret the spherical objects in the SEM micrographs to be particles of hydroxyapatite. Their appearance is similar to those reported in the literature^38^.

Energy dispersive X-ray spectroscopy (EDAX) analysis established the presence of hydroxyapatite (calcium and phosphate) in the mineralized paper scaffolds (Fig. 6b). EDAX provided the content of calcium and phosphorus in the samples in weight per cent (%). We then calculated the ratio of calcium to phosphate (Ca:P). The mineral composition was similar to that of the hydroxyapatite, with a Ca:P ratio of 1.5 ± 0.1 (n = 7). This ratio is consistent with physiologic deposition of hydroxyapatite by osteoblasts^25^,^27^. The minerals deposited by the same type of cells were previously reported to include poorly crystalline hydroxyapatite^39^. The Ca:P ratio of 1.5 ± 0.1 is also in agreement with that of the poorly crystalline hydroxyapatite^40^,^41^.

## Conclusions

This work demonstrates the use of paper to guide the deposition of mineral (calcium phosphate) by osteoblasts. It has the advantage of a biological read-out—mineralization— that is relatively easy to follow and quantify. The deposition of calcium phosphate in the 3D paper scaffolds described here are the first functional examples of template-guided mineralization by osteoblasts in a 3D system. Although in a previous report adipose-derived stem cells (ADSC) that were grown in polycaprolactone/gelatin electrospun nanofibrous membranes demonstrated regeneration of bone in a calvarial defect in a mouse model^42^, achieving mineralization by osteoblasts is a greater challenge due to their short life-span and possibility for de-differentiation.

Our approach uses a readily available material—paper—as the cell culture scaffold. Paper has a porous, fibrous structure, and enables transport of nutrient and O_2_ across its entire thickness. Paper, thus, supports a uniform distribution of cells on seeding (in collagen matrices), and allows cellular remodeling, proliferation, and differentiation of cells. Because paper is available in a wide range of thicknesses, chemical functionalities, and porosities, it allows for tuning of material properties and the construction of 3D tissue models. Paper can be easily patterned (e.g., by wax printing and by embossing) to control the distribution of cells in it. Paper is simply sterilized by soaking in ethanol, or autoclaving, and is also compatible with the growth of different types of cells other than osteoblasts^18^,^19^,^20^,^21^,^22^,^23^.

In addition to these advantages, paper scaffolds also have drawbacks. For instance, because of light scattering, paper that is thicker than 200 μm is difficult to image by fluorescent microscopy; as a result, it may be difficult to visualize the mineralized samples via fluorescent staining. Wet paper also has limited mechanical properties (strength, impact resistance). Without further development, simple paper sheets also limit the fabrication of more complex forms of tissue (e.g. vascularized tissue). In the current study we cultured osteoblasts to induce biomineralization; to explore formation of vascular bone tissue, we would need to carry out co-cultures with endothelial cells.

Taken together, our data suggests that the paper-based cell culture platform is a valuable system with which to study the processes involved in mineralization by osteoblasts. The samples can be evaluated by standard colorimetric assays, and also by microscopic and spectroscopic methods. Paper has the potential to tackle the limitations of the currently available scaffolds (metal, ceramic, polymer, composite) including accessibility, cost, porosity, tunability, rigidity, and ease of fabrication. Validation experiments in animal models must be performed, but these experiments are for future studies. One possible application of the work would be to guide and accelerate bone growth in patients having defects in skeletal bone, or in bone repair, by guiding growth using paper constructs seeded with (possibly patient-specific) cells.

## Materials and Methods

### Materials

We purchased Whatman 114 filter paper, glycerol-2-phosphate, ascorbic acid, alizarin red S and 4,6-diamidino-2-phenylindole (DAPI) from Sigma-Aldrich (St Louis, MO). We obtained the paraformaldehyde solution (16% (v/v)) from Electron Microscopy Sciences (Hatfield, PA). Mouse antibodies to Osteocalcin and Alexa 647-labeled antibodies to rabbit IgG were supplied by Abcam (Cambridge, MA). We purchased trypsin-EDTA, penicillin-streptomycin, phalloidin (Texas Red-X), fetal calf serum (FCS), fetal bovine serum (FBS), alpha-minimal essential medium (α-MEM) medium, and Dulbecco’s phosphate buffered saline (DPBS) from Invitrogen (Carlsbad, CA). We bought the OsteoImage kit from Lonza Walkersville Inc. (Walkersville, MD). We used all of the reagents as received without further purification.

### Preparation of Paper Scaffolds

Wax printing is a widely used procedure to generate patterns on paper. Abundant details for wax printing have been provided in the previous papers by both our group and others^20^,^21^,^22^,^43^,^44^,^45^. We prepared the free-standing scaffolds using Whatman 114 filter paper. We used these scaffolds to induce template-guided mineralization. Paper folding for different applications, generation of paper-based pop-up devices, and origami-inspired paper structures have been previously explored and details for fabrication procedures have been reported^46^,^47^,^48^,^49^,^50^. In our experiments, we manually manipulated filter paper to generate the folded structures. We created creases and then folded and rolled the paper to fabricate the 3D scaffolds. The diameter of different structures varied from 8 mm to 35 mm, and the length of the scaffolds was 25 mm. Scale bars were provided in Fig. 2 to indicate the dimensions of the scaffolds. For practical reasons, we used flat sheets of paper to carry out the biological assays and microscopy analyses. We performed wax printing with a commercial Xerox Phaser 8560 printer (Xerox Corporation, Norwalk, CT) to create hydrophobic boundaries and spatially defined hydrophilic seeding zones on Whatman 114 filter paper. We placed the filter paper in the wax printer and sent the print job from the Adobe Illustrator file in which we generated the patterns to define the cell seeding zones (diameter of each zone is 3 mm). When the printing job was complete, we took the wax-patterned paper from the printer and baked it for 60 sec at 150 °C for wax to melt and diffuse into the paper in 3D. The hydrophobic wax separated the cell seeding zones from each other. The paper scaffolds allow 10 samples to be cultured in parallel, and enable the growth of multiple replicates. We could have smeared the collagen cell suspension over the whole scaffold; however, in order to contain the samples within only defined zones of paper we carried out wax-printing.

Sterilization of the paper is critical before the cell-seeding step. We ensured the sterility of the paper scaffolds by soaking them in 90% (v/v) ethanol and drying them prior to seeding cells. We submerged the paper scaffolds in 90% (v/v) ethanol and incubated them for 30 min in a laminar air flow hood, and then air-dried them in the same hood under aseptic conditions. We used 90% grade ethanol, as it was the commercially available grade that we used in the laboratory. We could alternatively use other methods of sterilization including autoclaving, gamma irradiation, or ethylene oxide sterilization. Ethylene oxide has the potential to react with the surface of paper, autoclaving can alter shape and mechanical properties, and gamma irradiation is not universally available. Each method has strengths and weaknesses.

### Cell Cultures

We grew MLO-A5 osteoblasts in α-MEM that contained 5% FBS and 5% FCS. We kept the cell cultures in a standard 37 °C incubator equipped to provide 5% CO_2_. Upon preparation of the cell-laden paper scaffolds, we changed the culture medium to differentiation medium, which was α-MEM supplemented with 10% (v/v) FBS, ascorbic acid, and glycerol-2-phosphate. We changed the medium every other day to provide a fresh environment for cells.

We used a collagen gel as the matrix to support cell growth. Collagen is the most abundant extracellular matrix protein in bone^51^. Collagen type I is an essential component for mineralization of bone^25^. We used a collagen concentration of 2.5 mg/mL to embed the cells. Higher concentrations of collagen resulted in premature gelation; lower concentrations were too weak to remain in the pores of the paper matrix. The cells were suspended in a collagen hydrogel matrix and added to the paper. When gelation occurred, the cells were entrapped within the collagen hydrogel matrix in the paper scaffold in 3D.

In order to prepare 1 mL of collagen gel at a concentration of 2.5 mg/mL, we placed 800 μL collagen type I (initial concentration: 3.8 mg/mL), 100 μL α-MEM medium, and 100 μL 10X α-MEM medium into an eppendorf tube. Addition of 200 μL sodium hydroxide (initial concentration: 100 mM) into this mixture brought the pH to a neutral (pH 7.2–7.4) condition, which allowed gelation when the temperature of the gel was brought to 37 °C. The cells were suspended in the resulting collagen solution, added to the paper scaffolds, and placed in the growth medium at 37 °C. The constructs were maintained in the culture incubator for different time points up to 3 weeks.

We suspended the cells in a collagen matrix at different cell densities (0.1 × 10^6^, 0.4 × 10^6^, and 1.6 × 10^6^ cells per sample) and deposited 2 μL of the cell-laden gel solution into the cell-seeding zones of a wax-printed paper scaffold, or added 1 mL of the cell suspension (80 × 10^6^ cells per sample) in collagen to the folded 3D scaffolds.

### Immunocytochemistry

For the paper-based cell culture platform to be successful, we must demonstrate that the cells express specific markers of bone. To assess the phenotype of the cultured cells, we tested them for their expression of osteocalcin, which is a bone-specific protein. We fixed the samples in 4% (v/v) paraformaldehyde, and permeabilized the cells using 0.5% (v/v) Triton X-100. We used goat serum (10% v/v) to block non-specific binding. We then incubated the samples with 1/200 diluted primary antibody to osteocalcin overnight at 4 °C. We used the secondary antibodies in a 1/500 dilution ratio for 1 h. We performed DAPI staining following the manufacturer’s protocols. We also performed negative control experiments for osteocalcin staining using only the secondary antibody (no primary antibody). As expected, we did not observe non-specific binding of the secondary florescent antibody to the samples.

Similarly, we carried out fluorescent staining for hydroxyapatite according to the manufacturer’s procedure. We rinsed the samples three times with PBS and imaged them with a Zeiss LSM710 confocal microscope (Carl Zeiss Microscopy, LLC, Thornwood, NY).

### Characterization of Mineralized Samples

Alizarin red staining: At each time, we fixed the samples with 4% (v/v) paraformaldehyde. We freshly prepared a 2% (w/v) solution of alizarin red, and adjusted the pH to 4.2. We incubated the samples with the alizarin red solution for 20 min, washed and imaged them with an upright microscope. We quantified the intensity of red color from the images using NIH ImageJ.

Scanning electron microscopy (SEM) and energy dispersive X-ray spectroscopy (EDAX): The samples were fixed, frozen in liquid nitrogen, and lyophilized. These samples were then sputter-coated by palladium/platinum at a ratio of 80:20, and imaged at a beam accelerating voltage of 10 kV. We performed high-resolution imaging by Zeiss Supra55VP Field Emission SEM (Carl Zeiss Microscopy, LLC, Thornwood, NY) to visualize the presence of calcium and phosphate in the mineralized paper constructs.

Micro-Computed Tomography (Micro-CT): The cells were seeded in the paper scaffolds and cultured for 21 days. A Nikon Metrology (X-Tek) HMXST225 micro-CT system (Metris X-Tek, UK), which is equipped with an X-ray gun in the reflection mode was used to analyze the dry samples. Molybdenum (Mo) was used as the X-ray target in the experiments. Micro CT scans were performed by applying a voltage source at 85 kV and a current source at 95 μA. The reconstruction of scanned samples was performed using the software VGStudio MAX (Heidelberg, Germany). The mineralized areas on paper constructs appeared as a bright white color.

### Statistical Analysis

To analyze the experimental data, we used statistical software GraphPad Prism (Version 4.02, La Jolla, CA). We determined the statistical differences between groups by utilizing one-way ANOVA analyses. We considered the *p*-values < 0.05 as statistically significant (*p < 0.05, **p < 0.01, ***p < 0.001).

## Additional Information

**How to cite this article**: Camci-Unal, G. *et al*. Biomineralization Guided by Paper Templates. *Sci. Rep.* **6**, 27693; doi: 10.1038/srep27693 (2016).

## Supplementary Material

Supplementary Information
