# Supplementary material for: Biomineralization Guided by Paper Templates
Source: Sci Rep. 2016 Jun 9;6:27693. doi: 10.1038/srep27693 (PMC4899756; doi:10.1038/srep27693)
Supplement: Supplementary Information [file srep27693-s1.docx]

**Supplementary Information**

**Biomineralization Guided by Paper Templates**

Gulden Camci-Unal^1^, Anna Laromaine^2^, Estrella Hong^1^, Ratmir Derda^3^, and George M. Whitesides^1,4*^

^1^Department of Chemistry and Chemical Biology, Harvard University, 12 Oxford Street, Cambridge, MA 02138, USA.

^2^Institut de Ciència de Materials de Barcelona, ICMAB-CSIC, Campus UAB, Bellaterra, Catalunya, E-08193 Spain.

^3^Department of Chemistry, University of Alberta, Edmonton, Alberta, T6G 2G2, Canada.

^4^Wyss Institute for Biologically Inspired Engineering, Harvard University, 60 Oxford Street, Cambridge, MA 02138, USA.

(*) Author to whom correspondence should be addressed: gwhitesides@gmwgroup.harvard.edu

Keywords: Biomineralization; Osteoblasts; Bone; Paper

**Supplementary Information**

*Structure of bone*

Bone is a dynamic structure that continuously remodels throughout its lifetime^1^. Remodeling takes place in response to changes in biomechanical forces, mechanical injury, or to adapt the strength of the bone^2^. An injury to the bone initiates a cascade of complex processes that regenerate the damaged areas. The process of spontaneous healing begins with the formation of a hematoma (blood clot), and elicits an inflammatory response. The hematoma attracts immune cells via signaling molecules^3^. Fibroblasts subsequently migrate towards the site of the injury and lay down extracellular matrix (ECM), which is primarily composed of collagenous proteins (mainly collagen type I) and proteoglycans^4^. Deposition of matrix leads to the formation of a fibrous cartilage (callus, rich in collagen type I), which stabilizes the healing tissue mechanically. As the repair progresses, the callus progressively vascularizes and mineralizes into woven bone, which is eventually replaced by compact bone^3^.

Bone is composed—in addition to its solid hydroxyapatite structural elements—of four types of cells: osteoclasts, bone-lining cells (also known as osteoprogenitor cells), osteoblasts, and osteocytes^4^. Osteoclasts are multinucleated cells that are derived from macrophages. The primary function of osteoclasts is to digest bone by secreting acidic proteins and enzymes^1^. The bone-lining cells are of mesenchymal origin from the bone marrow and remain quiescent unless there is an external stimulus (mechanical, hormonal, and/or nutritional)^2^. The bone-lining cells turn into osteoblasts when the signaling molecules direct them to deposit bone minerals in response to an external factor (mechanical stimulation, microdamage, and/or injury)^1^. Osteoblasts are responsible for formation of bone by laying down collagenous matrix, which is subsequently mineralized by precipitation of calcium and phosphate^5^. During the process of mineralization, some of the osteoblasts are trapped and become buried inside the matrix; there, they terminally differentiate into osteocytes^2^. The osteocytes provide a structural network for the bone. In this work, we studied osteoblasts, and their deposition of minerals in structured scaffolds.

*Scaffolds for bone*

The ideal scaffold for bone must be porous, resilient, biodegradable, biocompatible, osteoinductive (enabling differentiation of cells into the bone lineage), and osteoconductive (enabling bone to grow on a surface)^6^. The most common scaffolds of bone include polymeric materials (e.g., poly (caprolactone) (PCL), poly(lactic-co-glycolic acid) (PLGA), poly(propylene fumarate) (PPF), polyurethane (PU), polyethylene glycol (PEG), gelatin, collagen, alginate, chitosan, silk, and starch), metals (e.g., stainless steel, platinum, titanium, and cobalt), and inorganic materials (e.g., hydroxyapatite and β-tricalcium phosphate (β-TCP))^7^. Composite materials have also been used to overcome limitations and improve the characteristics of single-material scaffolds^8^. Different materials, which can complement the features of each other, can be combined to control the properties of composite scaffolds such as degradation, biocompatibility, and osteointegration.

**Supporting Table S1.** Limitations of the conventional orthopaedic scaffolds for encapsulation of cells. Paper can be used to address these limitations.

| **Type of scaffold** | **Limitations** |
| --- | --- |
| Metals | Risk of infection^9^, allergic reactions^10^, corrosion^11^, release of toxic metal ions into the body^12^, failure of osseointegration^13^, loss of bone around implant^14^, high cost^15^, multi-step fabrication procedures^16^. |
| Polymers | Difficult to control porosity^17^, low mechanical strength^18^, lack of bioactivity^19^, not low-cost^20^, might degrade quickly^21^, toxic degradation products^22^, use of organic solvents^23^, prone to wear and tear^24^, poor processability^25^, multi-step fabrication procedures^23^. |
| Ceramics | Brittleness^26^, poor fracture toughness^27^, not resilient^28^, difficult to shape^29^, difficult to control porosity^30^, slow degradation rate^22^, failure of cellular ingrowth^31^, multi-step fabrication procedures^32^. |
| Composite materials | Difficulty in controlling/predicting degradation^33^, risk of toxicity from residual solvents^34^, non-uniform distribution of organic and inorganic phases^35^, difficulty in chemically binding the components^36^, difficult to shape^37^, not cost-effective^38^, multi-step fabrication procedures^32^. |
| Hydrogels | Weak mechanical integrity^20^, might be difficult to sterilize^39^, unstable functional groups^40^, difficulty in controlling degradation profile^41^, may be difficult to incorporate and retain bioactive functional groups^42^, large changes in volume due to shrinking and swelling^43^, multi-step fabrication procedures^44^. |
|  |  |
| ***PAPER* can be used to address the major limitations of conventional scaffolds. Paper is a widely available, low-cost, porous, flexible, and biocompatible material that is easy to sterilize.** | |

**Supporting Table S2.** Disadvantages of conventional scaffolds for bone.

| **Type of scaffold** | **Commercially available** | **Low-cost** | **Easy fabrication** | **Porosity is easy to control** | **Flexible** | **Does not release toxic products** | | **Mechanically strong** | **Biocompatible *in vivo*** |
| --- | --- | --- | --- | --- | --- | --- | --- | --- | --- |
| Metals | No^16^ | No^15^ | No^16^ | No^16^ | No | | No^12^ | Yes^45^ | Yes^46^ |
| Polymers | No^20^ | No^20^ | No^23^ | No^17^ | No | | No^22^ | No^18^ | Yes^46^ |
| Ceramics | No^32^ | Yes^32^ | No^32^ | No^30^ | No | | No^33^ | Yes^29^ | Yes^47^ |
| Composites | No^32^ | No^38^ | No^37^ | No^30^ | No | | No^34^ | Yes^28^ | No^48^ |
| Hydrogels | Yes^49^ | Yes^50^ | No^44^ | No^51^ | Yes | | No^52^ | No^20^ | Yes^42^ |
|  |  |  |  |  |  | |  |  |  |
| Paper | Yes^53^ | Yes^54^ | Yes^55^ | Yes^56^ | Yes^57^ | | Yes^58^ | Yes^53, 59-62^ | Yes^53^ |

**References**

1 Kini, U. & Nandeesh, B. N. in *Radionuclide and Hybrid Bone Imaging* (eds Fogelman Ignac, Gnanasegaran Gopinath, & Van Der Wall Hans) 29-57 (Springer 2012).

2 Clarke, B. Normal bone anatomy and physiology. *J. Am. Soc. Nephrol.* **3, Supplement 3**, S131-S139 (2008).

3 Sfeir, C., Ho, L., Doll, B. A., Azari, K. & Hollinger, J. O. in *Bone Regeneration and Repair: Biology and Clinical Applications* (eds J. R. Lieberman & G. E. Friedlaender) 21-44 (Humana Press Inc., 2005).

4 Rao, R. R. & Stegemann, J. P. Cell-based approaches to the engineering of vascularized bone tissue. *Cytotherapy* **15**, 1309-1322 (2013).

5 Neve, A., Corrado, A. & Cantatore, F. P. Osteoblast physiology in normal and pathological conditions. *Cell Tissue Res.* **343**, 289-302 (2011).

6 Venugopal, J., Low, S., Choon, A. T., Kumar, A. B. & Ramakrishna, S. Electrospun-modified nanofibrous scaffolds for the mineralization of osteoblast cells. *J. Biomed. Mater. Res. A* **85A**, 408-417 (2008).

7 Amini, A. R., Laurencin, C. T. & Nukavarapu, S. P. Bone tissue engineering: recent advances and challenges. *Crit. Rev. Biomed. Eng.* **40**, 363-408 (2012).

8 Murphy, M. B. *et al.* Multi-Composite Bioactive Osteogenic Sponges Featuring Mesenchymal Stem Cells, Platelet-Rich Plasma, Nanoporous Silicon Enclosures, and Peptide Amphiphiles for Rapid Bone Regeneration. *J. Funct. Biomater.* **2**, 39-66 (2011).

9 Chang, C. C. & Merritt, K. Infection at the site of implanted materials with and without preadhered bacteria. *J. Orthop. Res.* **12**, 526-531 (1994).

10 Amini, M. *et al.* Evaluation and management of metal hypersensitivity in total joint arthroplasty: a systematic review. *J. Long-Term Eff. Med. Implants* **24**, 25 (2014).

11 Böstman, O. & Pihlajamäki, H. Clinical biocompatibility of biodegradable orthopaedic implants for internal fixation: a review. *Biomaterials* **21**, 2615-2621 (2000).

12 Okazaki, Y. & Gotoh, E. Comparison of metal release from various metallic biomaterials in vitro. *Biomaterials* **26**, 11-21 (2005).

13 Parithimarkalaignan, S. & Padmanabhan, T. Osseointegration: An Update. *J. Indian Prosthodont. Soc.* **13**, 2-6 (2013).

14 Magone, K., Luckenbill, D. & Goswami, T. Metal ions as inflammatory initiators of osteolysis. *Arch. Orthop. Trauma Surg.* **135**, 683-695 (2015).

15 Mantripragada, V. P., Lecka‐czernik, B., Ebraheim, N. A. & Jayasuriya, A. C. An overview of recent advances in designing orthopedic and craniofacial implants. *J. Biomed. Mater. Res. A* **101**, 3349-3364 (2013).

16 Ryan, G., Pandit, A. & Apatsidis, D. P. Fabrication methods of porous metals for use in orthopaedic applications. *Biomaterials* **27**, 2651-2670 (2006).

17 Smith, I. O., Liu, X. H., Smith, L. A. & Ma, P. X. Nanostructured polymer scaffolds for tissue engineering and regenerative medicine. *Wiley Interdiscip. Rev.: Nanomed. Nanobiotechnol.* **1**, 226-236 (2009).

18 Sabir, M., Xu, X. & Li, L. A review on biodegradable polymeric materials for bone tissue engineering applications. *J. Mater. Sci.* **44**, 5713-5724 (2009).

19 G. Chen, T. Ushida, T. Tateishi, Scaffold Design for Tissue Engineering. *Macromolecular Bioscience* **2**, 67-77 (2002).

20 Liu, X. & Ma, P. Polymeric Scaffolds for Bone Tissue Engineering. *Ann. Biomed. Eng.* **32**, 477-486 (2004).

21 Karageorgiou, V. & Kaplan, D. Porosity of 3D biomaterial scaffolds and osteogenesis. *Biomaterials* **26**, 5474-5491 (2005).

22 Chen, Q., Zhu, C. & Thouas, G. Progress and challenges in biomaterials used for bone tissue engineering: bioactive glasses and elastomeric composites. *Prog. Biomater.* **1**, 1-22 (2012).

23 Hutmacher, D. W. Scaffolds in tissue engineering bone and cartilage. *Biomaterials* **21**, 2529-2543 (2000).

24 Bose, S., Roy, M. & Bandyopadhyay, A. Recent advances in bone tissue engineering scaffolds. *Trends Biotechnol.* **30**, 546-554 (2012).

25 Gunatillake, P. A. & Adhikari, R. Biodegradable synthetic polymers for tissue engineering. *Eur. Cells Mater.* **5**, 1-16 (2003).

26 Bucholz, R. W. Nonallograft osteoconductive bone graft substitutes. *Clin. Orthop. Relat. Res.* **395**, 44-52 (2002).

27 Zeeshan, S. *et al.* Biodegradable Materials for Bone Repair and Tissue Engineering Applications. *Materials* **8**, 5744-5794 (2015).

28 Brahatheeswaran, D., Yasuhiko, Y., Toru, M. & Kumar, D. S. Polymeric Scaffolds in Tissue Engineering Application: A Review. *Int. J. Polym. Sci.* **2011** Article ID 290602 (2011).

29 Du, D. *et al.* Microstereolithography-Based Fabrication of Anatomically Shaped Beta-Tricalcium Phosphate Scaffolds for Bone Tissue Engineering. *BioMed Res. Int.* **2015**, Article ID 859456 (2015).

30 Munch, E. *et al.* Porous ceramic scaffolds with complex architectures. *JOM* **60**, 54-58 (2008).

31 Rainer, B., Anika, J., Aurica, M., Jana, M. & Enrico, M. New Coating Technique of Ceramic Implants with Different Glass Solder Matrices for Improved Osseointegration-Mechanical Investigations. *Materials* **6**, 4001-4010 (2013).

32 Sáenz, A., Rivera-Muñoz, E., Brostow, W. & Castaño, V. M. Ceramic Biomaterials: An Introductory Overview. *J. Mater. Educ.* **21**, 297 - 306 (1999).

33 Wu, S., Liu, X., Yeung, K. W. K., Liu, C. & Yang, X. Biomimetic porous scaffolds for bone tissue engineering. *Mater. Sci. Eng.* **80**, 1-36 (2014).

34 Yang, S., Leong, K. F., Du, Z. & Chua, C. K. The design of scaffolds for use in tissue engineering. Part I. Traditional factors. *Tissue Eng.* **7**, 679 (2001).

35 Zhang, Y., Wu, C., Friis, T. & Xiao, Y. The osteogenic properties of CaP/silk composite scaffolds. *Biomaterials* **31**, 2848-2856 (2010).

36 Uskokovic, V. Nanostructured platforms for the sustained and local delivery of antibiotics in the treatment of osteomyelitis. *Crit. Rev. Ther. Drug Carrier Syst.* **32**, 1 (2015).

37 Patel, N. R. & Gohil, P. P. A Review on Biomaterials: Scope, Applications & Human Anatomy Significance. *IJETAE* **2**, 91-101 (2012).

38 Xigeng, M. & Dan, S. Graded/Gradient Porous Biomaterials. *Materials* **3**, 26-47 (2009).

39 Gkioni, K., Leeuwenburgh, S. C. G., Douglas, T. E. L., Mikos, A. G. & Jansen, J. A. Mineralization of hydrogels for bone regeneration. *Tissue Eng. Part B* **16**, 577 (2010).

40 Huaping, T. & Kacey, G. M. Injectable, Biodegradable Hydrogels for Tissue Engineering Applications. *Materials* **3**, 1746-1767 (2010).

41 Jia, X. & Kiick, K. L. Hybrid multicomponent hydrogels for tissue engineering. *Macromol. Biosci.* **9**, 140 (2009).

42 Hoffman, A. S. Hydrogels for biomedical applications. *Adv. Drug Delivery Rev.* **64**, 18-23 (2012).

43 Ionov, L. Biomimetic Hydrogel‐Based Actuating Systems. *Adv. Funct. Mater.* **23**, 4555-4570 (2013).

44 Billiet, T., Vandenhaute, M., Schelfhout, J., Van Vlierberghe, S. & Dubruel, P. A review of trends and limitations in hydrogel-rapid prototyping for tissue engineering. *Biomaterials* **33**, 6020-6041 (2012).

45 Kelly, A. & Hideo, N. Metallic Scaffolds for Bone Regeneration. *Materials* **2**, 790-832 (2009).

46 Agarwal, R. & García, A. J. Biomaterial strategies for engineering implants for enhanced osseointegration and bone repair. *Adv. Drug Delivery Rev.* **94**, 53-62 (2015).

47 Christel, P. *et al.* Biomechanical Compatibility and Design of Ceramic Implants for Orthopedic Surgery. *Ann. N. Y. Acad. Sci.* **523**, 234-256 (1988).

48 Ignatius, A. A., Betz, O., Augat, P. & Claes, L. E. In vivo investigations on composites made of resorbable ceramics and poly(lactide) used as bone graft substitutes. *J. Biomed. Mater. Res.* **58**, 701-709 (2001).

49 Jones, A. & Vaughan, D. Hydrogel dressings in the management of a variety of wound types: A review. *J. Orthop. Nurs.* **9**, S1-S11 (2005).

50 Cao, L. *et al.* Bone regeneration using photocrosslinked hydrogel incorporating rhBMP-2 loaded 2-N, 6-O-sulfated chitosan nanoparticles. *Biomaterials* **35**, 2730-2742 (2013).

51 Chiu, Y.-C., Larson, J. C., Isom, A. & Brey, E. M. Generation of porous poly(ethylene glycol) hydrogels by salt leaching. *Tissue Eng., Part C* **16**, 905-912 (2010).

52 Nicodemus, G. D. & Bryant, S. J. Cell encapsulation in biodegradable hydrogels for tissue engineering applications. *Tissue Eng., Part B* **14**, 149-165 (2008).

53 Derda, R. *et al.* Paper-supported 3D cell culture for tissue-based bioassays. *Proc. Natl. Acad. Sci. U.S.A.* **106**, 18457-18462 (2009).

54 Mosadegh, B. *et al.* Three-Dimensional Paper-Based Model for Cardiac Ischemia. *Adv. Healthc. Mater.* **3**, 1036-1043 (2014).

55 Mosadegh, B. *et al.* A paper-based invasion assay: Assessing chemotaxis of cancer cells in gradients of oxygen. *Biomaterials* **52**, 262-271 (2015).

56 Derda, R. *et al.* Multizone Paper Platform for 3D Cell Cultures. *PLoS One* **6**, e18940 (2011).

57 Camci-Unal, G., Newsome, D., Eustace, B. K. & Whitesides, G. M. Fibroblasts enhance migration of human lung cancer cells in a paper-based co-culture system. *Adv. Healthc. Mater.* DOI: 10.1002/adhm.201500709 (2015).

58 Deiss, F. *et al.* Platform for High-Throughput Testing of the Effect of Soluble Compounds on 3D Cell Cultures. *Anal. Chem.* **85**, 8085-8094 (2013).

59 Lichtblau D. *et al.* Determination of mechanical properties of historical paper based on NIR spectroscopy and chemometrics – a new instrument. *Appl. Phys. A* **92**, 191-195 (2008).

60. Espy, H. H. The mechanism of wet-strength development in paper: A review. *TAPPI J.* **78**, 90-99 (1995).

61. Roberts, J. C. in *Chemistry of Paper 1st edn* Ch. 4, 59-65 (Royal Society of Chemistry, 1996).

62. Hamedi, M. M. *et al.* Electrically Activated Paper Actuators. *Adv. Funct. Mater.* DOI: 10.1002/adfm.201505123 (2016).

**Supplementary Figures**

**
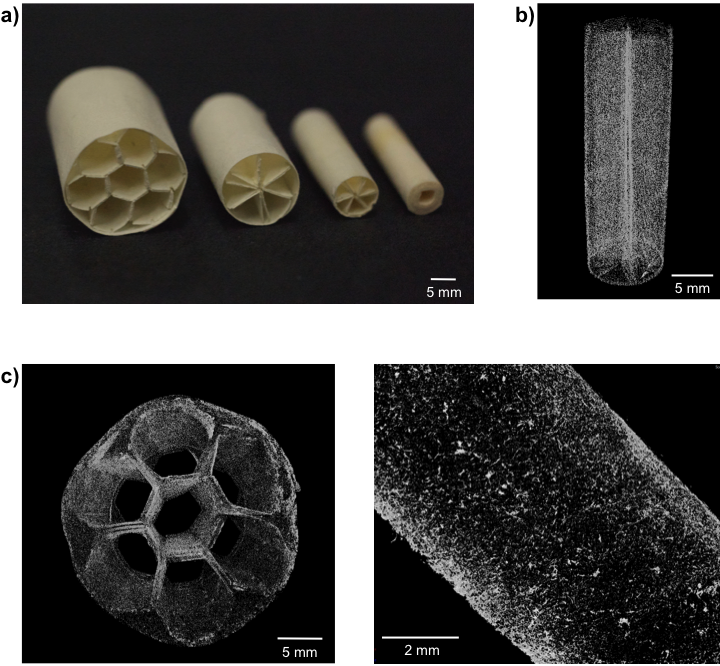
**

**Figure S1.** Biomineralized origami-inspired paper scaffolds. a) The cells were seeded in the paper scaffolds and cultured for 21 days. b-c) The micro-CT X-Ray scans illustrated the mineralized areas in the paper constructs in bright white color.

**
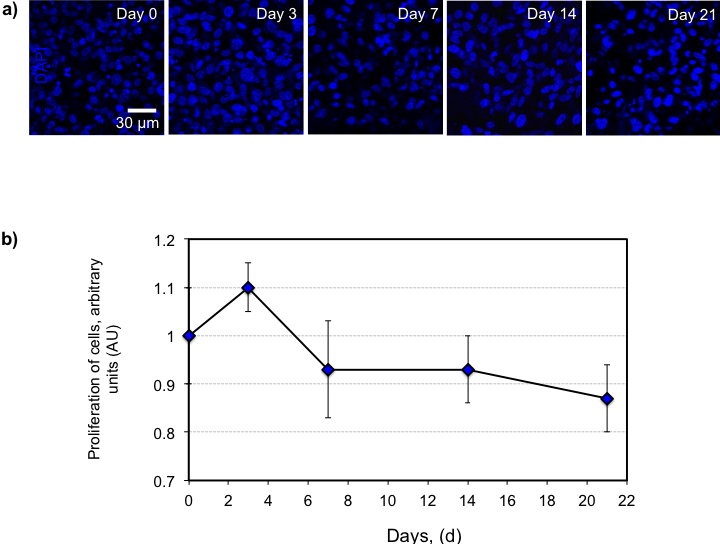
**

**Figure S2.** Proliferation of cells in the collagen matrix in paper scaffolds at different time points. We stained the nuclei of the cells to image the distribution and proliferation of the cells on days 0, 3, 7, 14, and 21. The initial seeding density was 1.6x10^6^ cells/sample. We stained the samples with DAPI (blue), and obtained the images by confocal microscopy. The results indicated that proliferation increased until day 3 and then decreased after day 7. Because proliferation slows down at the onset of mineralization, this result is expected. The scale bar represents 30 μm.


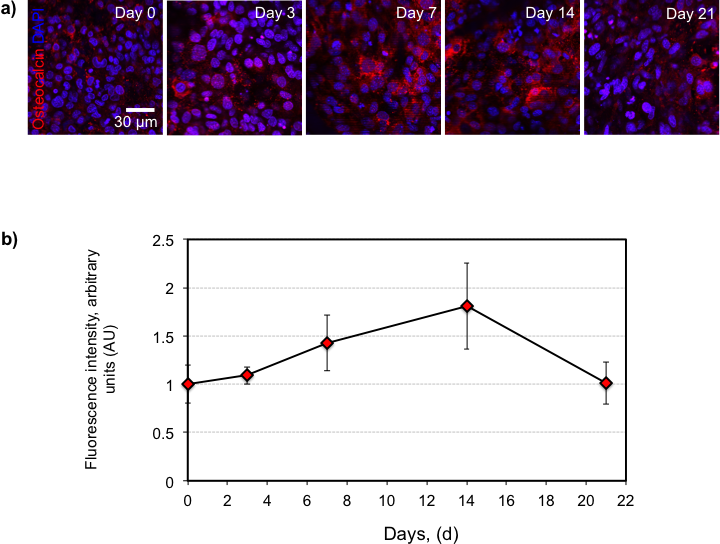


**Figure S3.** Expression of a bone-specific marker, osteocalcin, was determined by immunocytochemistry in the paper scaffolds. The initial cell density was 1.6x10^6^ cells/sample. We carried out immunostaining for osteocalcin (red) on days 0, 3, 7, 14, and 21, and acquired the fluorescent images by confocal microscopy. We counter-stained the cells with DAPI (blue) to visualize the nuclei of the cells. The expression of osteocalcin increased until day 14 and then decreased. This result could be due to increasing mineralization after day 14. The scale bar represents 30 μm.
